# Navigating the Digital Storm: A Systematic Review and Meta-Analysis of Technology-Induced Job Insecurity, Its Outcomes and Moderators

**DOI:** 10.3390/bs16091474

**Published:** 2026-08-25

**Authors:** Jinghan Liang, Chung-Hong Wong

**Affiliations:** School of Psychological and Cognitive Sciences, Peking University, Beijing 100871, China; w39356623@163.com

**Keywords:** technology-induced job insecurity, systematic review, meta-analysis, employee, cognitive attitudes, affective states, behavioral responses, moderators, stress appraisal theory, threat rigidity theory

## Abstract

As automated technology advances rapidly, human employees’ fear of being replaced (i.e., technology-induced job insecurity) has become a widespread stress in today’s workplace. It is crucial for organizations to recognize this new technological challenge and address its impact appropriately. Therefore, to systematically understand its impacts and explore potential buffering factors, the present research conducts a systematic review and meta-analysis of 96 studies (*N* = 43,104 individuals). Drawing on the tripartite response model, we classify their outcome variables into three broad categories encompassing nine subgroups. Furthermore, integrating stress appraisal theory and threat-rigidity theory, we examine two critical moderators reflecting specific coping capability against technological threats. Our findings indicate that technology-induced job insecurity is systematically associated with employees’ cognition, affection, and behavior. Notably, the moderation analysis highlights distinct boundary conditions under which these associations vary across different outcomes, emphasizing the complexity of these effects. Specifically, low threat proximity—providing a crucial time buffer before the actual threat happens—is associated with weaker negative attitudes toward the organization, and lower withdrawal and resistance behaviors. Individuals’ high learning ability—reflecting strong adaptive flexibility—is associated with weaker negative appraisals of the job itself and more sustained proactive behaviors. In summary, the present research contributes to a systematic understanding of how technology-induced job insecurity is related to human psychology and behaviors at work, thereby offering valuable insights to inspire future research and management practice.

## 1. Introduction

**Figure 1:**
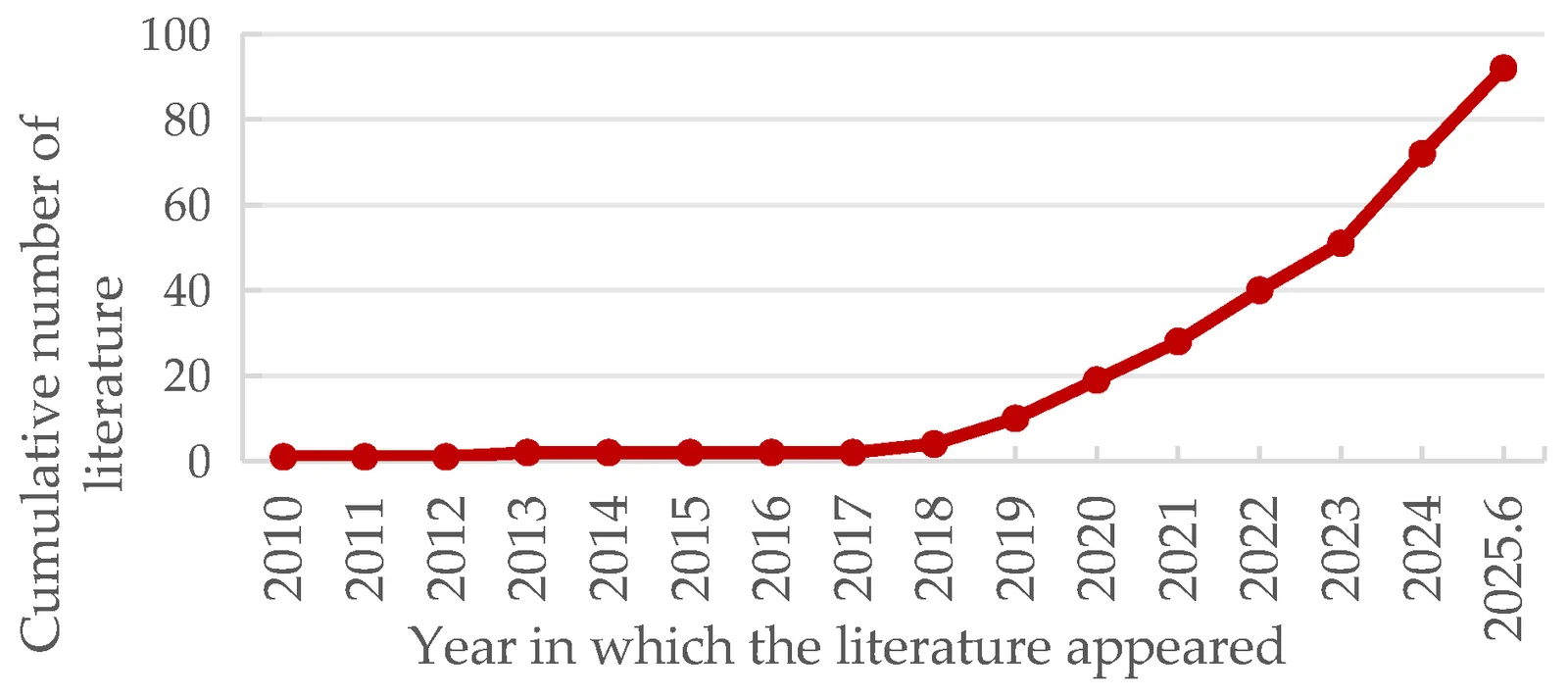
The included literature on technology-induced job insecurity and work-related outcomes.

The development of automated technology has significantly improved work efficiency across various industries, yielding unprecedented productivity increases for organizations. However, the overwhelming efficiency advantage of automation has made employees increasingly aware that their roles may not be irreplaceable. This awareness has been associated with growing technology-induced job insecurity (Nam, 2019; Walczok & Bipp, 2025; Yam et al., 2023). The latest AI Index Report (Maslej et al., 2025) from Stanford University reveals that over 60% of employees today are facing technological changes in their jobs and expressing high levels of replacement anxiety. Due to the prevalence of this emerging workplace stress, the issue has rapidly attracted considerable interest from the academic community (see Figure 1). Existing research has thoroughly examined the negative impacts of technology-induced job insecurity on employees’ psychological and behavioral responses, including lower job satisfaction, diminished organizational identification, and withdrawal or resistant behaviors toward new technologies, etc. (S. Bai et al., 2024; Chiu et al., 2023; Yam et al., 2023; J. Zhao et al., 2023). These negative impacts inevitably impede organizations’ digital transformation. Consequently, both scholars and practitioners increasingly recognize that such insecurity may be difficult to avoid. This is especially the case given the rapid and continuing pace of technological advancement. Instead, the focus must quickly shift to finding effective ways to buffer against it (Jiang, 2025).

According to stress appraisal theory, perceiving a situation as a threat typically triggers stress and negative behavioral responses. A key factor in buffering these effects is how individuals evaluate their coping capability (Lazarus, 1991; Lazarus & Folkman, 1984). Although prior studies have examined potential moderators from this perspective, no clear consensus has emerged. This lack of consensus may be attributed to two critical limitations. On one hand, the existing literature is highly fragmented, with inconsistent terminology. Researchers across disciplines often use different terms to refer to the core concept of technology-induced job insecurity, reflecting the classic “jangle fallacy” (see Table 1), which refers to using seemingly different variables to capture a similar construct (Block, 1995; Jiang, 2025; Oreg et al., 2011). Moreover, prior studies have examined highly diverse outcome variables, but these variables have not been organized into a clear overarching framework (see Table 2). On the other hand, many studies have examined moderators from typical coping-resource-based perspectives. However, they often overlook the distinctive nature of technology-induced job insecurity as a novel form of stress. Specifically, it undermines employees’ sense of self-worth and future career prospects, and such insecurity is chronic and continually evolving (Koen & Parker, 2020; Probst et al., 2025). As a result, many traditional resources—such as training focused on a single skill or relying on self-efficacy built on past successes—tend to yield only limited effects in practice (Hampel et al., 2022; Koen & Parker, 2020; Lingmont & Alexiou, 2020).

These limitations raise two key questions. First, which types of interventions are most likely to show buffering associations? Second, are these patterns consistent across different outcomes? Without an integrative framework to synthesize existing findings, further empirical studies may add confusion rather than insight for scholars and practitioners. To overcome these limitations, we first develop a systematic framework for reviewing the existing literature. Using this framework, we synthesize evidence accumulated over the past two decades. Meanwhile, we examine whether the relationships between technology-induced job insecurity and work-related outcomes vary as a function of moderating factors, and whether such moderating patterns differ across outcomes. Methodologically, we use a meta-analytic approach to test our hypothesis, thereby strengthening the robustness of our findings and providing empirically grounded insights for future interventions.

## 2. Literature Review

### 2.1. Conceptualization of Technology-Induced Job Insecurity

One of the earliest conceptualizations of technology-induced job insecurity, termed “techno-insecurity”, was introduced by Tarafdar et al. (2007). It highlights situations in which people feel threatened by losing their jobs due to the adoption of new technologies, extending the traditional concept of job insecurity into the technological domain. With the rapid development of automated technology and its growing potential to replace human labor, research interest in this unique form of work stress has surged sharply since 2019 (see Figure 1), resulting in an increasingly diverse range of terms and descriptions (see Table 1). These terms can range from explicit words (e.g., techno-insecurity) to vague phrases (e.g., perceived substitution crisis). Some terms focus on employees’ cognitive awareness of the replacement risk (e.g., STARA awareness), while others focus on employees’ emotional states (e.g., affective automation-related job insecurity). This jangle of terms reflects growing scholarly concern with this topic. However, it has also contributed to mixed findings among scholars and practical challenges for practitioners.

Fortunately, our summary (see Table 1) reveals that despite the diversity of terms, this research shares a common core component in definitions: individuals appraise technology as a threat to their current jobs. More importantly, most scales used to measure this concept are adapted from well-established scales of job insecurity (C. Lee et al., 2018; Shoss, 2017), further confirming that they are indeed discussing the same phenomenon. That is to say, there exists a problematic trend of “jangle fallacy”. To address the fallacy and facilitate clearer discussion in the following sections, we propose a unified term “technology-induced job insecurity”. We define it as individuals’ subjective perception of technological threats to their job continuity. By focusing on the consistency of the core component rather than the terminology, we can identify more relevant research and integrate their findings.

### 2.2. Using the Tripartite Response Model to Organize Individuals’ Reaction to Technology-Induced Job Insecurity

Our review identified a wide range of outcome variables examined in this literature. These variables range from commonly studied constructs, such as self-efficacy, to highly specific and context-dependent outcomes, such as violations of information security policy (see Table 2). While this diversity reflects the richness of research focus in this field, it often overlooks the fact that many seemingly different variables essentially capture the same core aspect of individuals’ psychology and behavior. For example, variables such as self-efficacy, self-esteem, and status, though phrased differently, all essentially reflect individuals’ self-evaluation at work. Similarly, while resistance to technology and innovation resistance differ in the level of detail they convey, both reflect the same category of behavioral responses. Therefore, to systematically synthesize existing findings and develop our hypotheses, we introduce the tripartite response model to integrate fragmented outcome variables.

The tripartite response model is rooted in the tripartite view of attitudes in social psychology and has been adapted to explain employees’ multifaceted responses to organizational change and specific organizational initiatives (Kanitz et al., 2024; Oreg et al., 2011; Piderit, 2000). It conceptualizes individuals’ responses as comprising cognitive, affective, and behavioral dimensions, thereby capturing what employees think, feel, and do in relation to the same focal object. Drawing on this perspective, the present research applies the tripartite response model to the context of technological change and uses it to classify employees’ responses to technology-induced job insecurity. This application offers two main advantages. First, the framework reflects the fundamental structure of individuals’ psychological and behavioral responses. Its broad applicability therefore facilitates the integration of the diverse and fragmented outcome variables examined in prior research. Second, and more importantly, the framework is designed to capture differences across response dimensions toward the same focal object. It thus provides a natural basis for distinguishing the differential associations of technology-induced job insecurity with cognitive, affective, and behavioral outcomes, as well as the potentially distinct boundary conditions underlying these associations. In this way, the framework helps prevent an overly simplified understanding of the consequences of technology-induced job insecurity.

Building on the tripartite response model, we further divide these broad categories into several theoretically grounded subgroups (see Table 2). This classification is also aligned with established paradigms for job insecurity outcomes in organizational behavior research and helps capture subtle differences among variables (C. Lee et al., 2018; Shoss, 2017). Variables within the broad category of cognition can be split into three subgroups. Self-evaluation reflects individuals’ beliefs about their capabilities, attitudes toward jobs reflect individuals’ assessment of the job’s value, and attitudes toward organizations reflect the assessment of the organization’s trustworthiness. Although they differ in referents, together they reflect individuals’ cognitive thoughts when faced with job insecurity. Similarly, variables within the broad category of affection can also be split into two subgroups. Some variables reflect individuals’ immediate psychological strain (e.g., job strain), while others reflect individuals’ long-term affective well-being outcomes resulting from prolonged stress exposure (e.g., job burnout). They respectively reflect how individuals feel in the short and long term when faced with work stress (Bliese et al., 2017). In addition, variables within the broad category of behavior require a more detailed classification, as individuals’ reactions to job insecurity are highly complex and cannot be adequately captured by a simple positive–negative taxonomy (Shoss, 2017; Shoss et al., 2023). For example, even within the scope of negative behaviors, overt resistance (e.g., counterproductive work behavior) significantly differs from passive disengagement (e.g., quiet quitting) in terms of the level of activation. Therefore, drawing on the more established two-dimensional taxonomy (valence and activation), we classify behavioral outcomes into four subgroups (Kanitz et al., 2024; Oreg et al., 2018). Basic task performance reflects a state of low-activation work engagement, where individuals only fulfil their in-role duties. Proactive behavior reflects a state of high-activation work engagement, where individuals make self-initiated and extra-role contributions. Withdrawal behavior reflects a state of low-activation oppositional response, where individuals reduce work engagement and passively avoid challenges. Resistance behavior is a state of high-activation oppositional response, where individuals display fierce opposition or even destructive actions. We group them under the same broad category and collectively name them behavioral responses.

### 2.3. Relationships Between Technology-Induced Job Insecurity and Work-Related Outcomes

Since technology-induced job insecurity is a specific type of work stress, we selected the dominant stress appraisal theory in this field as our theoretical framework to develop hypotheses (Jiang, 2025; Shoss, 2017). Stress appraisal theory emphasizes that individuals’ responses are not directly triggered by objective environmental stimuli but rather by their subjective appraisal of those stimuli. In the appraisal process, an environmental stimulus can be appraised as either an opportunity or a threat. When appraised as a threat, individuals may anticipate future losses and therefore exhibit a series of negative responses (Lazarus, 1991; Lazarus & Folkman, 1984). In line with this theory, the connotation of technology-induced job insecurity implies a consensus that individuals have already appraised new technology as a threat (see Table 1; Hampel & Sassenberg, 2021; B. J. Kim & Kim, 2024; J. Li et al., 2019; Turja et al., 2022), which suggests that further discussion of its impacts should be conducted within the threat-appraisal framework.

Regarding the impacts on individuals’ cognitive attitudes, technology-induced job insecurity can cause harm in several aspects. First, previous research has shown that individuals’ self-evaluation will be negatively affected by the overwhelming efficiency advantage of automated technology over human labor. Employees may feel a strong sense of insecurity about whether their abilities and skills will remain valued in the future (Goethals & Ziegelmayer, 2022; Vorobeva et al., 2022; Yam et al., 2023). Second, individuals’ attitudes toward their jobs will be negatively impacted, as their jobs could be replaced by cold, impersonal technology. Employees may perceive the job as meaningless and lacking a future (Buzas et al., 2025; Brougham & Haar, 2018; Granulo et al., 2019). Additionally, individuals’ attitudes toward the organization may be negatively affected. Although technology-induced job insecurity originates largely from external technological developments, when employers actively adopt technologies that substitute for human labor, or fail to safeguard employees’ job security during technological disruption (Baser et al., 2025; Hampel & Sassenberg, 2021; J. Li et al., 2019), the organization may be perceived as unreliable (S. Bai et al., 2024; Brougham & Haar, 2018; J. Zhao et al., 2023).

**Hypothesis** **1.** *Technology-induced job insecurity is negatively related to employees’ cognitive attitudes, including self-evaluation (H1a), attitudes toward job (H1b), and attitudes toward organization (H1c).*

Regarding the impact on individuals’ affective states, there is no doubt that technology-induced job insecurity can cause greater harm than any other form of work stress. This is because individuals can hardly compete with automated technology in terms of efficiency, nor can they stop the increasing trend of using technology in the workplace (Vorobeva et al., 2022; Yam et al., 2023). Consequently, employees are likely to experience intense immediate psychological strain (Girardi et al., 2024; Jin et al., 2024; La Torre et al., 2024; H. J. Yang et al., 2025). Even worse, because of the lack of solutions to resolve this tension, they can only endure continuous exposure to the work environment with high psychological strain, which will finally drain their mental resources and damage their long-term affective well-being (Jin et al., 2024; Pericleous et al., 2025; H. J. Yang et al., 2025; Zheng & Zhang, 2025). As a result, we hypothesize as follows.

**Hypothesis** **2.** *Technology-induced job insecurity is negatively related to employees’ affective states. Specifically, it will increase their immediate strain (H2a) and reduce long-term affective well-being (H2b).*

Regarding individuals’ behavioral responses to technology-induced job insecurity, by integrating threat-rigidity theory into stress appraisal theory, we argue that employees typically exhibit a negative, conservative bias. Specifically, threat-rigidity theory is a well-established framework for explaining how individuals and organizations respond to external threats. Its core premise is that when faced with a significant external threat, individuals tend to narrow their information processing and instinctively revert to their most familiar and habitual behavioral patterns (i.e., “rigidity”). Even if these habitual behavioral patterns are no longer effective for adapting to change, they help decrease the uncertainty caused by change and protect the individual’s current resources as much as possible (Staw et al., 1981; Mazzei et al., 2025). Based on this reasoning, individuals’ behavioral responses will manifest in two ways. On one hand, positive work behaviors, including in-role work effort and extra-role proactivity, are likely to decline. Employees may reduce their in-role work effort (i.e., basic task performance) because, in a job-threatening situation, individuals’ primary goal shifts to survival, which requires only meeting baseline performance standards rather than pursuing high performance as usual (Gupta & Banerjee, 2026). Even if employees intend to maintain their previous level of high performance, they may lack sufficient resources to do so, as their cognitive and emotional resources are diverted toward processing threat-related information rather than being allocated to task completion (Hart & Cooper, 2001; Probst et al., 2025; Staw et al., 1981). Similarly, engaging in extra-role proactive behaviors is also unwise at this time because it requires investing additional resources and taking on extra risk. Not to mention, the narrowing of their cognitive focus limits their ability to demonstrate flexible, extra-role behaviors (Bolino et al., 2010; Parker et al., 2019; Y. Yang et al., 2024). On the other hand, employees might increasingly show withdrawal and resistance. These responses are not only a clear sign of behavioral rigidity but also a hidden way to conserve resources, helping them maintain a sense of psychological safety amid the uncontrollable wave of technological change. For example, by avoiding new technology, employees can maintain their habitual work routines without investing extra effort in learning and adapting to new work processes (Beaudry & Pinsonneault, 2005; Gupta & Banerjee, 2026; Pirkkalainen et al., 2019; C. Yang & Jiang, 2025; X. Zhao et al., 2020). Furthermore, employees may exhibit counterproductive behaviors, such as deliberately disrupting technical procedures, to prove their irreplaceability within the organization. This helps them maintain their jobs in the short term, in other words, protect the most valuable resource (i.e., the job itself) from loss (Beaudry & Pinsonneault, 2005; Hampel & Sassenberg, 2021; D. G. Kim & Lee, 2021). To summarize, we develop the following research hypotheses.

**Hypothesis** **3.** *Technology-induced job insecurity is negatively related to employees’ basic task performance (H3a) and proactive behavior (H3b).*

**Hypothesis** **4.** *Technology-induced job insecurity is positively related to employees’ withdrawal behavior (H4a) and resistance behavior (H4b).*

### 2.4. Moderating Roles of Technological Threat Proximity and Individuals’ Learning Ability

The stress appraisal theory suggests that how people evaluate their coping capability—including both external environmental support and internal abilities—plays a significant role in buffering the adverse effects of threats and shaping future responses (Lazarus, 1991; Lazarus & Folkman, 1984; Oreg et al., 2018). In the past, many studies followed this theory to identify resource-oriented solutions. However, the unique nature of technology-induced job insecurity means that many traditional resources may no longer effectively support individuals’ coping. For example, traditional skills training is a common external support intervention. Nevertheless, given the rapid pace of technological change, routine training programs targeting specific skills quickly become outdated, fail to provide long-term solutions, and are therefore limited in their effectiveness at addressing ongoing technological threats (Hampel et al., 2022; Lingmont & Alexiou, 2020). Furthermore, organizations struggle to survive and adapt in high-uncertainty environments driven by technological advancements, which also hinder their ability to provide employees with timely, ongoing training support (Bhattacharya & Wright, 2005; Cascio & Montealegre, 2016). Another example is an internal trait like self-efficacy. While it is viewed as an important coping resource, automated technologies often surpass human capabilities, thereby greatly undermining self-efficacy (Goethals & Ziegelmayer, 2022; Vorobeva et al., 2022; Yam et al., 2023). Therefore, viewing self-efficacy as a universal solution in fact overlooks the realities of technological change. In summary, based on this reflection, our research goes beyond the typical resource framework and explores potential moderating variables specifically related to the unique nature of technology-induced job insecurity, examining what truly alleviates its psychological harm and behavioral rigidity (Mazzei et al., 2025; Jeong et al., 2023; Hootegem et al., 2023).

On one hand, when facing the overwhelming impact of automation, an intense sense of urgency serves as the core situational trigger for behavioral “rigidity.” High urgency deprives individuals of the time needed to gather information, evaluate alternative solutions, and adapt to change, leaving them with insufficient coping options (Beck & Schmidt, 2013; Carstensen et al., 1999; Hardy et al., 2024). Feeling powerless to alter the situation, employees instinctively shift their focus toward short-term personal resource protection, inevitably retreating into rigid behavioral patterns (Jiang, 2025; Oreg et al., 2018). For example, when individuals experience job insecurity but perceive themselves as low-status and with limited coping options, they may choose to remain silent about problems to avoid investing more effort and to avoid potential conflict (Son et al., 2023). Therefore, we introduce the concept of threat proximity, defined in the previous job insecurity literature as how soon an expected threat will occur. Unlike job insecurity (which emphasizes the subjective perception of the degree of threat), this concept focuses more on the urgency of the threat (Shoss et al., 2023). Thus, we propose that when employees face technology-related job insecurity, a lower threat proximity—indicating the threat is distant—provides a time cushion, potentially reducing its harmful impact.

**Hypothesis** **5.** *The negative relationships between technology-induced job insecurity and work-related outcomes are weaker when technological threat proximity is lower.*

On the other hand, because technology-induced job insecurity is chronic—characterized by continual skill devaluation—single-skill training is ineffective. Instead, a person’s core learning ability serves as the primary internal resource for addressing behavioral rigidity. Learning ability, representing a fundamental form of adaptive flexibility and agility (DeRue et al., 2012; Lyndgaard et al., 2024; Noe et al., 2014), determines how swiftly employees recover from technological disruptions, such as performance declines (Hardy et al., 2024; Lang & Bliese, 2009) and self-doubt (DeRue et al., 2012; Lyndgaard et al., 2024). Rather than falling into rigidity, individuals with high learning ability are well equipped to adapt to new challenges and respond proactively (Bednall & Sanders, 2017; Lyndgaard et al., 2024; Niessen, 2006). Therefore, we argue that high learning ability functions as the crucial internal buffer against the adverse effects of technology-induced job insecurity.

**Hypothesis** **6.** *The negative relationships between technology-induced job insecurity and work-related outcomes are stronger when the individuals’ learning ability is lower.*

**Figure 2:**
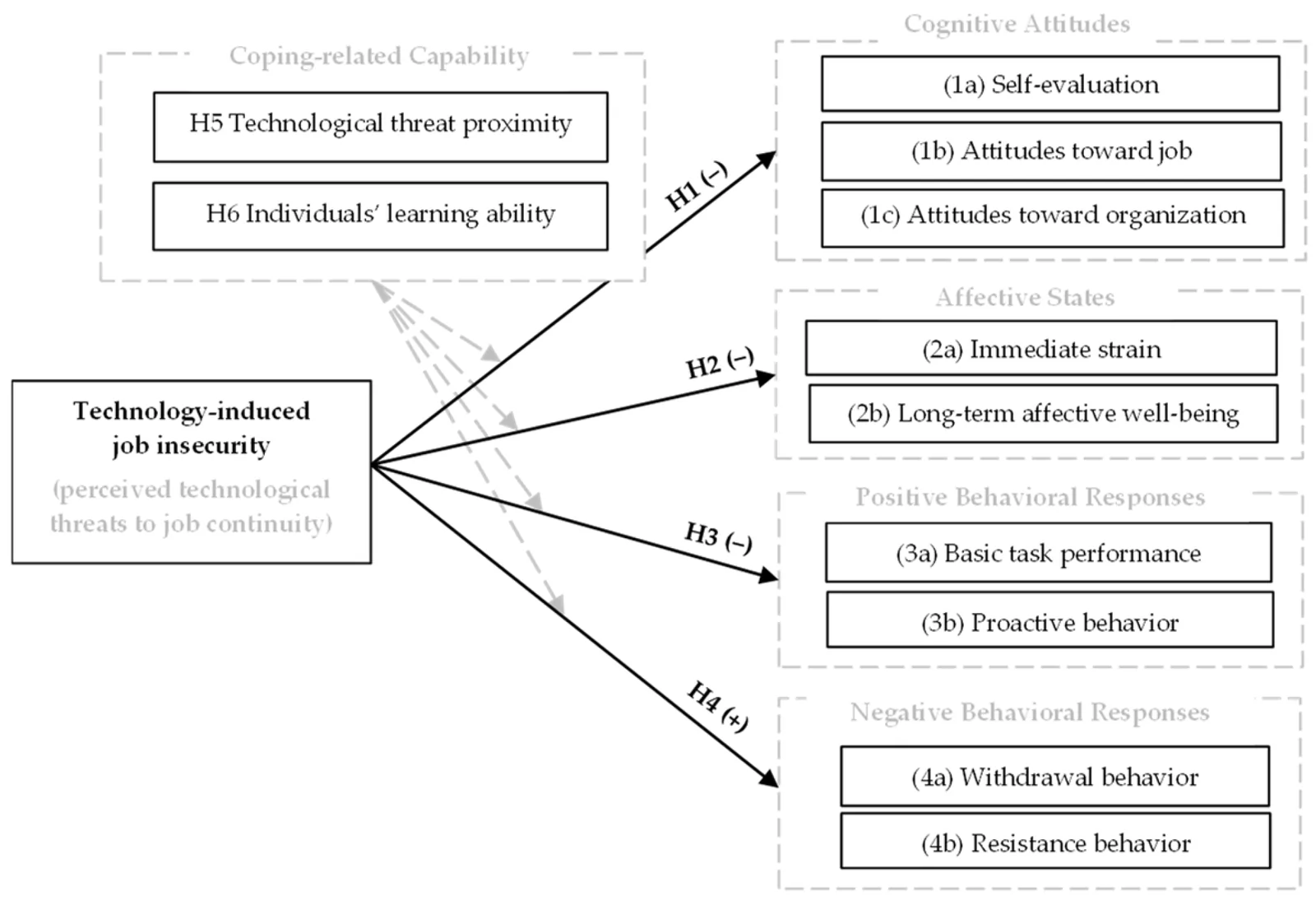
Research model.

Taken together, this study integrates two critical moderating variables that reflect an individual’s underlying capability to cope with technological threats, which completes our overarching conceptual framework (see Figure 2), which is then examined using a meta-analytic approach.

## 3. Materials and Methods

**Figure 3:**
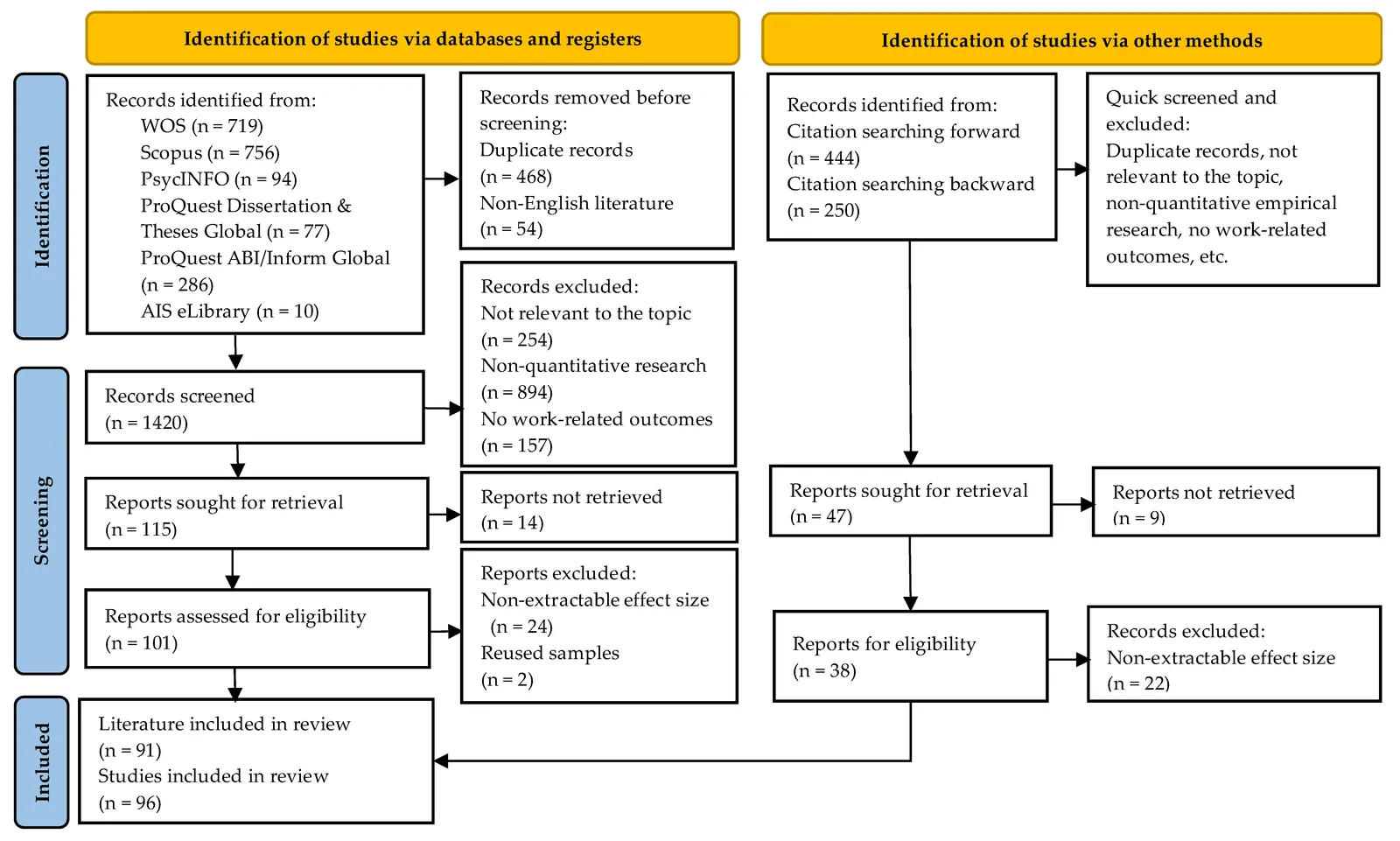
PRISMA flow diagram of literature search and inclusion process (Page et al., 2021).

The review was conducted in accordance with the PRISMA (Preferred Reporting Items for Systematic Reviews and Meta-Analyses) checklist (see File S1 in Supplementary Materials). To find all relevant research on technology-induced job insecurity, we conducted a systematic literature search following the PRISMA flow (see Figure 3) across multiple databases (including Web of Science, Scopus, PsycINFO, ProQuest ABI/Inform Global, and AIS eLibrary) for peer-reviewed manuscripts published between January 2000 and June 2025. The search targeted literature containing the pre-selected keywords (“technology-induced job insecurity” OR “technology-based job insecurity” OR “technophobia” OR “smart technology, artificial intelligence, robotics, and algorithms awareness” OR “STARA awareness” OR “job automating technology awareness” OR “artificial intelligence and robotics awareness” OR “fear of replacement” OR “perceived substitution crisis” OR “technological unemployment” OR “artificial intelligence system automation” OR “affective automation-related job insecurity”) in their title or abstract. We completed this search with several supplementary strategies, including a backward search of the reference lists of recent reviews on this topic (Nastjuk et al., 2023; Yuan et al., 2025) and a forward search of citations to highly cited papers on this topic (Brougham & Haar, 2018). In addition, to identify unpublished studies, we searched the ProQuest Dissertations & Theses database and some conference proceedings.

### 3.1. Inclusion Criteria and Sample

To be included in this meta-analysis, the study needed to meet the following criteria (see Figure 3): (a) provide a full text written in English; (b) be empirical and quantitative; (c) be conducted among employees in work context; (d) measure technology-induced job insecurity at the individual level; (e) measure at least one work-related psychological or behavioral outcome; and (f) report enough information for effect size extraction. When we encountered research that conducted more than one study, we treated them as independent samples to extract the effect size. When multiple studies reported data from the same sample, only one was included in the meta-analyses to avoid double-counting. In accordance with these criteria, the two authors conducted the screening process independently. Weekly meetings were held to address any discrepancies. This process ultimately resulted in 91 studies (96 independent samples, 43,104 individuals).

### 3.2. Variable Coding

#### 3.2.1. Dependent Variables Coding

Regarding the coding of dependent variables, we classified outcomes into three broad categories and nine subgroups (see Table 2). These classifications were initially made independently by two authors, with any discrepancies resolved through discussion. When extracting effect sizes, we chose correlation as the most appropriate summary statistic because it accurately estimated the true construct-level relationship between technology-induced job insecurity and outcomes. If correlations were not reported, alternative statistics (e.g., standardized regression coefficients) were used after appropriate transformation. To ensure that all effect sizes within a category were oriented in the same direction, we reverse-coded some when necessary. When a study included more than one dependent variable that could be coded into the same category, we combined them into a single composite effect size for analysis (Borenstein et al., 2009). The first and second authors independently coded all the studies. The kappa coefficients for initial coding comparisons were all above 0.80, indicating good inter-coder reliability. Afterward, the coded results were compared, and any significant discrepancies were resolved by a third party (a research assistant). The coded details for each study were provided (see File S2 in Supplementary Materials).

#### 3.2.2. Moderators Coding

We operationalized the moderators by developing a coding scheme firmly grounded in their conceptual definitions. At the same time, we considered the methodological requirements of meta-analysis, which call for both practical extractability and strong cross-study comparability. We ultimately adopted study- and sample-level proxy indicators, which can balance theoretical validity with empirical feasibility. Specifically, technological threat proximity captures the time employees have before a technological change directly affects their work. Past studies usually assess this urgency by considering how likely a job is to be automated (Koen & Parker, 2020). Essentially, the easier a role is to automate, the sooner the threat arrives. Following this logic, we objectively coded this variable using the Generative AI Exposure Index Scheme (Gmyrek et al., 2025). This scheme classifies different occupations based on exposure risk levels (1 = negligible risk, 6 = critical risk). Higher scores indicate greater objective vulnerability to technological disruption, in other words, a higher level of technological threat proximity. We matched the representative occupations of the sample in each empirical study to one of the classifications. When studies included mixed occupational samples or did not report occupational details, we coded them as missing values.

Regarding another moderator, learning ability reflects how quickly and flexibly a person can acquire new knowledge and skills. As education level is commonly used as a standard indicator of learning ability (Bednall & Sanders, 2017; Marchiori et al., 2020; Niessen, 2006), we objectively measured this variable by calculating the percentage of participants with a bachelor’s degree or higher in each study. A higher percentage of degree holders indicates a stronger average learning ability within that group. When studies did not report the educational level of samples, we coded them as missing values. The process for checking coding consistency and resolving discrepancies is the same as described above.

### 3.3. Meta-Analytic Procedures

We conducted our meta-analyses using the psychmeta package in R 4.5.1. We reported sample-size-weighted correlations (r) and true score correlations (ρ) after correcting for measurement error (Schmidt & Hunter, 2015). We reported 95% confidence interval (CI) to indicate the precision of the mean effect size estimate, and 80% credibility interval (CV) to describe the distribution of true effects across studies. In addition, we conducted visual assessment of funnel plots and Egger regression asymmetry tests to assess publication bias. We also conducted leave-one-out sensitivity analyses to assess the robustness of the findings.

## 4. Results

### 4.1. The Main Relationship

As shown in Table 3, the results largely confirmed our hypotheses, indicating that such insecurity is systematically negatively related to employees’ cognition, affection, and behavior. Regarding cognitive attitudes, technology-induced job insecurity was significantly related to employees’ self-evaluations (*ρ* = −0.14, 95% CI = [−0.22, −0.05], supporting H1a) and to job attitudes (*ρ* = −0.41, 95% CI = [−0.52, −0.29], supporting H1b). Regarding affective states, technology-induced job insecurity was not only positively related to immediate strain (*ρ* = 0.41, 95% CI = [0.24, 0.58], supporting H2a) but also negatively related to long-term affective well-being (*ρ* = −0.33, 95% CI = [−0.40, −0.26], supporting H2b). Finally, regarding behavioral responses, technology-induced job insecurity was negatively related to basic task performance (*ρ* = −0.18, 95% CI = [−0.29, −0.06], supporting H3a), positively related to more withdrawal (ρ = 0.34, 95% CI = [0.23, 0.46], supporting H4a) and resistance behaviors (*ρ* = 0.38, 95% CI = [0.24, 0.52], supporting H4b).

Interestingly, the main relationships between technology-induced job insecurity and organizational attitudes (*ρ* = −0.04, 95% CI = [−0.51, 0.59], not supporting H1c), and proactive behaviors (*ρ* = −0.05, 95% CI = [−0.19, 0.08], not supporting H3b) were not significant. This indicates that although technology-induced job insecurity is generally detrimental, its specific relationships may vary with key boundary conditions. This idea is also supported by meta-analytic distribution patterns: even for outcomes with notably negative main effects, the 80% credibility intervals remain quite broad, indicating significant variability in the percentage of true variance. These findings suggest that the effects of technology-induced job insecurity should not be understood as uniformly negative. Rather, these associations are complex and highly contingent on contextual boundary conditions, making it necessary to take moderating variables into account.

Overall, the above meta-analytic results demonstrated strong robustness and the absence of severe publication bias. Funnel plots and Egger’s tests (*p* > 0.05) showed no significant asymmetry across studies (see File S3 in Supplementary Materials). Leave-one-out sensitivity analyses confirmed that no single study disproportionately influenced the overall findings (after removing each study in turn, the overall mean effect size remained highly stable, with none of the 95% confidence intervals crossing zero).

### 4.2. The Moderation Analyses

**Figure 4:**
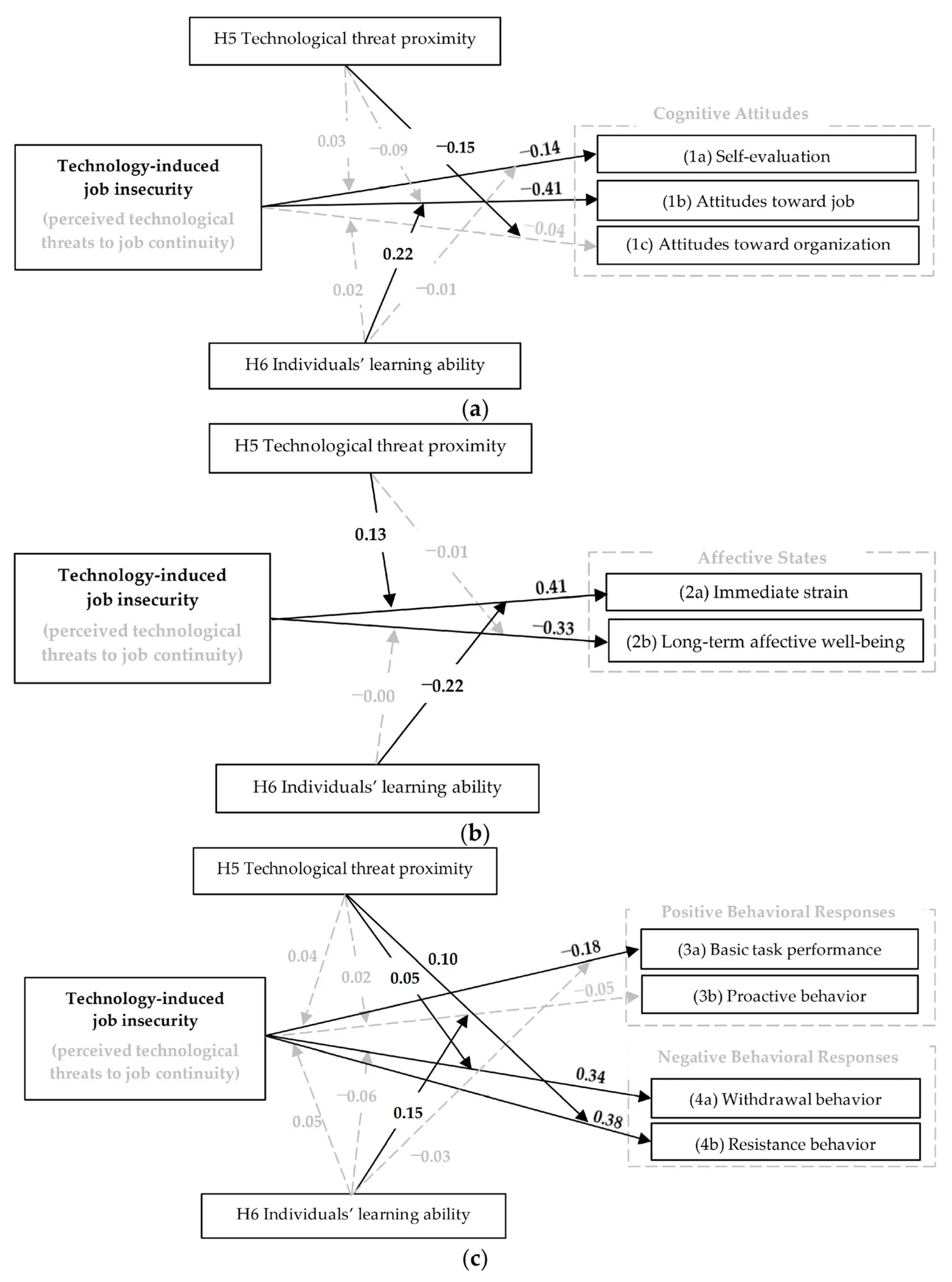
(**a**) Relationship between technology-induced job insecurity and cognitive attitudes. (**b**) Relationship between technology-induced job insecurity and affective states. (**c**) Relationship between technology-induced job insecurity and behavioral responses. Dashed lines indicate non-significant path coefficients.

We further examined the moderating roles of technological threat proximity and individuals’ learning ability (see Table 4, Figure 4). First, both moderators were associated with variations in the relationship between technology-induced job insecurity and affective outcomes, but these moderating patterns appeared to be temporally bounded. Specifically, the positive relationship between technology-induced job insecurity and immediate strain was significantly weaker under conditions of low technological threat proximity (*b* = 0.13, 95% CI = [0.00, 0.25]) and high learning ability (*b* = −0.22, 95% CI = [−0.38, −0.06]). In contrast, neither moderator significantly moderated the relationship between technology-induced job insecurity and long-term affective well-being (*b* = −0.01, 95% CI = [−0.08, 0.05]; *b* = −0.00, 95% CI = [−0.09, 0.09]). These findings suggest that the proposed moderating patterns were not uniform across outcomes. Rather, they varied by outcome category, indicating that differentiating among outcome categories is both meaningful and necessary.

Furthermore, subsequent meta-regression analyses highlighted distinct moderating patterns for the two moderators. On the one hand, external technological threat proximity was mainly related to associations involving organizational attitudes and defensive reactions. Specifically, low technological threat proximity was associated with a weaker negative relationship between technology-induced job insecurity and attitudes toward the organization (*b* = −0.15, 95% CI = [−0.29, −0.01]). It was associated with weaker positive associations between technology-induced job insecurity and withdrawal behavior (*b* = 0.05, 95% CI = [0.00, 0.10], H4a), and resistance behavior (*b* = 0.10, 95% CI = [0.00, 0.20]). On the other hand, individuals’ learning ability was mainly related to associations involving job-focused responses. Specifically, high learning ability was associated with a weaker negative relationship between technology-induced job insecurity and attitudes toward the job (*b* = 0.22, 95% CI = [0.05, 0.38]) and with a less negative relationship between technology-induced job insecurity and proactive behavior (*b* = 0.15, 95% CI = [0.01, 0.29]).

Moreover, although hypotheses 5 and 6 were generally supported, our findings showed that the effects of technology-induced job insecurity on certain outcomes were not significantly moderated by the variables proposed in this research. Beyond the aforementioned long-term affective well-being, these outcomes also included self-evaluation and basic task performance. These results suggest that technology-induced job insecurity may cause particularly deep harm to these domains. Future research needs to explore alternative and more effective buffering resources.

## 5. Discussion

In summary, this study develops an integrated framework for understanding the relationship between technology-induced job insecurity and various outcome variables, as well as the boundary conditions of these relationships. Most importantly, this framework enables examination of distinct moderating patterns for two types of coping conditions—external buffering and internal empowerment—across different outcomes. Specifically, low technological threat proximity and individuals’ high learning ability both appeared to moderate the negative relationship between technology-induced job insecurity and work-related outcomes, but they showed distinct moderating patterns. Low technological threat proximity appeared to reflect an external-buffering pattern: it was associated with more favorable perceptions of the organization and lower levels of withdrawal and resistance behaviors. Learning ability appeared to reflect an internal-empowerment pattern: it was associated with more positive cognitive appraisals of work value and more proactive coping behaviors. Overall, these findings move beyond traditional views that oversimplify the influence of technology-induced job insecurity and clarify the complexity of its relationship with work-related outcomes. Furthermore, the complexity of this phenomenon suggests that future interventions may require coordinated, multidimensional efforts.

### 5.1. Theoretical Implications

The primary theoretical contribution of this research is identifying the distinct mechanisms through which technology-induced job insecurity affects different types of outcome variables. As technological change accelerates, scholars have increasingly focused on the potential negative consequences of technology-induced job insecurity and have called for more targeted intervention research (Jiang, 2025). However, relevant findings remain fragmented and lack systematic integration and differentiation, leading to technology-induced job insecurity being broadly understood as a negative phenomenon, with no clear consensus on interventions. To address this issue, this research developed a more systematic analytical framework that integrates existing studies and shows that its effects are more complex than a simple, uniformly harmful view would suggest. Specifically, our findings show that the identified moderators can effectively buffer immediate affective strain but fail to mitigate longer-term affective impairment. This pattern highlights the theoretical value of distinguishing between short-term and long-term affective outcomes, as the fact suggests that these two forms of outcomes are governed by different coping logics: short-term resilience depends more on individual coping capabilities, whereas long-term recovery may rely more on broader systemic resources. In addition, our results show that different behavioral outcomes arise through distinct mechanisms. Reduced proactive behavior is mainly associated with weakened positive cognitive appraisals of one’s work value, whereas withdrawal and resistance are more closely linked to declining organizational trust. Taken together, these findings demonstrate the value of distinguishing among different categories of outcomes and reveal the complexity of the effects of technology-induced job insecurity, establishing a deeper, more systematic understanding of the novel technological phenomenon.

In addition, this study extends stress appraisal theory and threat-rigidity theory to the digital work context. Recent research frequently notes that traditional resources (e.g., past work experience, general self-efficacy) often fail to buffer the negative effects of technology-induced job insecurity (Hampel et al., 2022; Koen & Parker, 2020; Lingmont & Alexiou, 2020). Our study clarifies that this phenomenon is not a failure of the theories themselves, but rather because many traditional resources are poorly matched to the specific nature of technological threats. When automated technology directly threatens the value of accumulated experience and existing routines (Goethals & Ziegelmayer, 2022; Vorobeva et al., 2022; Yam et al., 2023), traditional interventions that emphasize current resources (which are already damaged) may no longer provide sustainable solutions. By situating technology-induced job insecurity in a more concrete context and identifying buffers that better match its core characteristics, this study deepens our understanding of which coping resources remain effective under sustained technological disruption and, therefore, extends the application of these classic theories.

### 5.2. Practical Implications

By identifying two specific moderators that mitigate the negative impacts of technology-induced job insecurity, our study offers concrete, dual-pathway guidance for management practice. First, organizations should avoid creating false urgency. Although lower perceived threat proximity helps employees maintain a long-term orientation and engage in proactive coping, some organizations mistakenly intensify the perceived urgency of technological replacement to “motivate” employees’ skill development (Jiang, 2025). This strategy is counterproductive, as it encourages employees to prioritize short-term resource preservation. Building on this consensus, we recognize that, in the context of rapid technological change, the actual time available for preparation is often limited. Even so, organizations can adopt alternative strategies to reduce such as phased technology adoption (Iansiti & Nadella, 2022; Parker & Grote, 2022). Such an approach supports a more gradual transition and provides a temporal cushion, enabling employees to respond more proactively.

Second, training programs should shift from training specific skills to fostering foundational learning ability. Given that prior research has shown that single-skill training is often insufficient in the face of ongoing technological change (Lyndgaard et al., 2024; Noe et al., 2014), our research explores and confirms that individuals’ learning ability provides a much stronger safeguard against ongoing technological changes. Put differently, knowing how to learn has become more important than knowing what to learn. Although developing such fundamental learning ability is more difficult than improving single work skills, it can still be fostered through well-designed interventions. For example, organizations can deliberately integrate cognitive training into job roles (Parker et al., 2021) or build a comprehensive learning ecosystem that provides multilevel support—offering immediate feedback on knowledge acquisition, reinforcing motivation at the meso level, and facilitating skill transfer at the macro level (Lyndgaard et al., 2024; Neeley & Leonardi, 2022).

### 5.3. Limitations and Future Directions

While this research offers a systematic synthesis of the literature, it has certain limitations that warrant further improvement and study. First, most of the studies we included rely on cross-sectional data, and we also used sample-level proxies to represent the moderator. This combination means our findings may indicate preliminary associations rather than causal conclusions. That said, this limitation is largely inherent to the meta-analytic approach, whose primary objective is to offer a study-level synthesis and preliminary validation of the existing literature—not to establish causality. Accordingly, our results are best understood as a systematic integration of correlational patterns within that literature, rather than definitive causal evidence. By clarifying the overall landscape, the present research lays the groundwork for more targeted future investigations. We therefore recommend that future research adopt longitudinal or experimental methods to strengthen causal understanding.

Second, the moderators we examined in the current study are relatively broad—for instance, individuals’ overall learning ability—meaning that some potentially critical emerging variables have yet to be incorporated into the analysis of moderating effects. Taking more proximal, technology-specific competencies as an example, digital literacy may possess stronger explanatory power (Gómez, 2020; Z. Chen & Zainudin, 2024; Choi et al., 2023; Farrell et al., 2021): it can help employees reduce uncertainty and regain a sense of control, thereby enabling them to view technological demands as more manageable challenges (Chan et al., 2021; Kadhim, 2024). However, empirical studies that simultaneously address the two constructs of technology-induced job insecurity and digital literacy remain extremely scarce at present, falling far short of the volume of literature required for a meta-analytic test of moderating effects. This makes it impossible for us to effectively discern, at a quantitative level, the unique buffering role played by this more targeted competency. In light of this, we suggest that future research continue to accumulate and enrich empirical evidence regarding the buffering effects of individual technology-specific competencies—such as digital literacy—against technological disruption.

Third, although we identified two core moderators, they did not significantly reduce the negative relationship between technology-induced job insecurity and self-evaluation, long-term affective well-being, or actual task performance. This may be because this form of job insecurity reflects a chronic, fundamental disruption that affects individuals’ psychological functioning and routine work processes. Relying solely on individual coping capabilities is unlikely to be sustainable in the long run, especially as personal resources become depleted over time. We therefore recommend that future research examine broader, structural interventions at the organizational level, as digital transformation is by no means an individual responsibility alone. For example, scholars could investigate human–technology augmentation work design and continuous reskilling infrastructure and adopt longitudinal designs to track their effects. Moreover, whether an organization, on the whole, fosters a climate conducive to transformation—such as leadership support and a psychosocial safety climate—that provides employees with a safe learning environment is equally critical (Montreuil et al., 2025) and warrants attention in future research. In sum, it is necessary to systematically examine the moderating effects of organizational-level factors, thereby more fully revealing the resource coordination mechanisms through which individuals and organizations jointly cope with the impact of technology.

## 6. Conclusions

The growing prevalence of automated technology in today’s workplace has significantly heightened job insecurity among human employees. A systematic review is therefore needed to fully understand this phenomenon and effectively manage its negative consequences. Using a meta-analytic approach, we synthesize fragmented findings from the relevant literature and reveal how this form of insecurity is related to multiple aspects of employees’ psychology and work behavior. On this basis, we highlight the roles of two key moderators that are closely tied to the distinctive demands of coping with this emerging challenge. In summary, the present research offers a theoretically grounded and empirically validated framework to advance future research and develop targeted interventions.

## Figures and Tables

**Table 1:** Commonly used terms and definitions of technology-induced job insecurity.

Term	Definition	Sample Item and Related Article	Source of the Measurement
Techno-insecurity	Employees may *fear job loss* or a decline in status due to new *digital technologies*.	I feel constant threat to my job security due to new technologies (Alshammary & Hilmi, 2024; Chiu et al., 2023; Ficapal-Cusí et al., 2025; Issa, 2022; L. Li & Wang, 2021; Mangundu & Mayayise, 2023; Shadbad & Biros, 2022; Tarafdar et al., 2007; Urukovicova et al., 2023; H. J. Yang et al., 2025).	We first identified a broad list of items that create technostress from the existing literature; techno-insecurity is associated with situations where users *feel threatened about losing their jobs* as a result of a new ICT replacing them.
Technology-based job insecurity	Technology-based job insecurity is defined as the powerlessness to maintain the desired status quo in *a job-threatening situation*—applying the more general concept of job insecurity to the field of *technology adoption*.	The robot could replace me as a worker (Hampel et al., 2022; Hampel & Sassenberg, 2021).	Items were derived from *previous research on job insecurity*.
AI-induced job insecurity	This phenomenon indicates the perceived *threat to job stability and continuity* due to the rapid advancement and integration of *AI technologies* in the workplace.	Even if I want to, I will not be able to keep my present job due to the adoption of artificial intelligence (B. J. Kim & Kim, 2024).	To measure AI-induced job insecurity, we adapted a scale originally from the *job insecurity scale.*
Affective automation-related job insecurity	We define affective automation-related job insecurity (AAJI) as employees’ affective appraisal (concern, worry, anxiety) of *STARA-driven task and job substitution*.	I fear that smart technology, artificial intelligence, robotics and automation will take over the majority of my current work (Walczok & Bipp, 2025).	One author generated 26 new items based on *the definitions of cognitive and affective job insecurity* and the aforementioned *STARA Awareness.*
The perception of job insecurity caused by robots	Job insecurity, defined as the threat of losing one’s work; this measures the perception of *job insecurity caused by robots*.	I fear that someday I will lose my job to a robot (Erebak & Turgut, 2021).	This scale measures the perception of *job insecurity* caused by robots.
Perceived substitution crisis	The substitution crisis triggered by augmented intelligence, which refers to *concerns of being replaced by the target technology*.	I think that augmented intelligence would likely to replace me in the future (Fan et al., 2020; Verma & Singh, 2022; P. Wang et al., 2025).	Four items are designed in consideration of possible crisis in being replaced, independent, *unemployed* and negatively impacted in diagnosis capacity.
The threat of technological unemployment	The fear of technological unemployment refers to *the threat of losing a job*, working hours, or income due to technical progress through which *machines replace human labor*.	I fear that robots will replace or reduce my work (Turja et al., 2022).	A single item reflects the likelihood of an individual becoming *unemployed* due to technological factors.
STARA awareness	STARA awareness captures the extent to which an employee views the likelihood of *Smart Technology, Artificial Intelligence, Robotics and* Algorithms *impacting their future career prospects*.	I think my job could be replaced by STARA (Brougham & Haar, 2018; Hur & Shin, 2024; Kang et al., 2023; Olya et al., 2024; C. Yang & Jiang, 2025; Yin et al., 2024; J. Zhao et al., 2023; Y. Yang et al., 2024).	STARA awareness was measured using four items developed for this study, based on *the job* *insecurity measure.*
AI and robotics awareness	AIRA is a subjective sense of how an employee considers *the threat of AI and robotics*, which is not exactly the same as objective AI and robotics.	I think my job has a high risk of bowing to automation and will be replaced by machines with AI (Caglar & Taskin, 2023; J. Li et al., 2019; H. Wang et al., 2022).	Four items were taken and modified from Brougham and Haar’s (2018) study to better align them within the context of hotels.
AI awareness	AI awareness refers to the extent to which individuals *perceive AI as replacing their occupation* or affecting the future of their profession.	I think the jobs related to my field of study will be replaced by AI (S. Bai et al., 2024; Y. Li & Geng, 2025; Zheng & Zhang, 2025).	We used four items Brougham and Haar (2018) developed to measure employee AI awareness.

*Note.* Italicized terms represent the core conceptual elements identified across the definitions of technology-induced job insecurity in the reviewed literature.

**Table 2:** Brief summary of the three categories of outcome variables.

Category	Subgroup and Specific Variable			
Cognitive attitudes	*Self-evaluation* self-esteem self-efficacy computer self-efficacy core self-evaluation creative self-efficacy status	*Attitudes toward job* meaningful work intrinsic motivation career satisfaction job satisfaction	*Attitudes toward organization* organizational commitment affective commitment psychological contract breach	
Affective states	*Immediate strain* job stress work stress job strain psychological strain performance pressure role stress biological indicators	*Long-term affective well-being* job burnout work exhaustion fatigue positive emotions negative emotions anxiety depression work affective well-being psychological well-being mental health psychological health psychological safety attachment security		
Behavioral responses	*Basic task performance* work engagement work performance productivity security compliance role-prescribed service	*Proactive behavior* active learning job crafting innovative work behavior techno-adoption behavior intention proactive service promotive voice	*Withdrawal behavior* turnover quiet quitting avoidance job crafting	*Resistance behavior* individual deviance organizational deviance counter-productivity behavior innovation resistance resistance to change knowledge hiding prohibitive voice violate security policy

*Note.* Italicized terms indicate the broader outcome categories, whereas the non-italicized terms indicate the specific variables included within each category.

**Table 3:** Meta-analytic results: the impacts of technology-induced job insecurity on work-related outcomes.

Variable	*k*	*N*	*r*	*ρ*	*SD_ρ_*	95% CI	80% CV	%VAR
Cognitive attitudes								
Self-evaluation	18	5969	−0.11	−0.14	0.16	[−0.22, −0.05]	[−0.35, 0.08]	13.42%
Attitudes toward job	16	13,979	−0.35	−0.41	0.22	[−0.52, −0.29]	[−0.70, −0.11]	2.34%
Attitudes toward organization	7	2029	0.03	−0.04	0.59	[−0.51, 0.59]	[−0.81, 0.89]	1.43%
Affective states								
Immediate strain	15	7502	0.35	0.41	0.30	[0.24, 0.58]	[0.00, 0.82]	2.40%
Long-term affective well-being	31	11,857	−0.27	−0.33	0.18	[−0.40, −0.26]	[−0.57, −0.09]	8.83%
Behavioral responses								
Basic task performance	21	8557	−0.15	−0.18	0.24	[−0.29, −0.06]	[−0.50, 0.15]	5.34%
Proactive behavior	28	9883	−0.05	−0.05	0.35	[−0.19, 0.08]	[−0.51, 0.40]	3.28%
Withdrawal behavior	16	5897	0.18	0.34	0.21	[0.23, 0.46]	[0.06, 0.63]	6.94%
Resistance behavior	14	5196	0.33	0.38	0.23	[0.24, 0.52]	[0.07, 0.70]	5.61%

*Note. k* = number of independent samples cumulated; *N* = cumulative sample size; *r* = sample-size-weighted correlation, not corrected for measurement errors; *ρ* = sample-size-weighted correlation corrected for measurement error; *SDρ* = standard deviation of *ρ*; 95% CI = confidence interval; 80% CV = credibility interval. %VAR = percentage of observed variance in correlations attributable to statistical artifacts.

**Table 4:** (**a**) Moderation results: Technological threat proximity as moderator. (**b**) Moderation results: Individuals’ learning ability as moderator.

**(a)**								
**Variable**	***k***	***b***	***SE***	***z***	***p***	**95% CI**	***R* ^2^**	***Q_M_***
IV → Cognitive attitudes								
IV → Self-evaluation	8	0.03	0.10	0.31	0.76	[−0.16, 0.22]	0.00%	0.09
IV → Attitudes toward job	9	−0.09	0.09	−0.94	0.35	[−0.27, 0.09]	0.00%	0.89
IV → Attitudes toward organization	5	−0.15	0.07	−2.11	0.04	[−0.29, −0.01]	50.55%	4.44 *
IV → Affective states								
IV → Immediate strain	10	0.13	0.06	2.02	0.04	[0.00, 0.25]	26.14%	4.06 *
IV → Long-term affective well-being	23	−0.01	0.03	−0.35	0.72	[−0.08, 0.05]	0.00%	0.13
IV → Behavioral responses								
IV → Basic task performance	18	0.04	0.05	0.82	0.42	[−0.06, 0.15]	0.00%	0.66
IV → Proactive behavior	19	0.02	0.06	0.33	0.74	[−0.10, 0.15]	0.00%	0.11
IV → Withdrawal behavior	11	0.05	0.02	2.04	0.04	[0.00, 0.10]	27.94%	4.15 *
IV → Resistance behavior	7	0.10	0.05	2.02	0.04	[0.00, 0.20]	37.68%	4.06 *
(**b**)								
**Variable**	***k***	***b***	***SE***	***z***	***p***	**95% CI**	***R* ^2^**	***Q_M_***
IV → Cognitive attitudes								
IV → Self-evaluation	10	−0.01	0.08	−0.10	0.92	[−0.16, 0.14]	0.00%	0.01
IV → Attitudes toward job	7	0.22	0.08	2.58	0.01	[0.05, 0.38]	62.29%	6.67 **
IV → Attitudes toward organization	6	0.02	0.14	0.15	0.88	[−0.25, 0.29]	0.00%	0.02
IV → Affective states								
IV → Immediate strain	10	−0.22	0.08	−2.73	0.01	[−0.38, −0.06]	42.66%	7.43 **
IV → Long-term affective well-being	20	−0.00	0.05	−0.04	0.97	[−0.09, 0.09]	0.00%	0.00
IV → Behavioral responses								
IV → Basic task performance	15	−0.03	0.07	−0.46	0.64	[−0.18, 0.11]	0.00%	0.21
IV → Proactive behavior	19	0.15	0.07	2.03	0.04	[0.01, 0.29]	15.53%	4.13 *
IV → Withdrawal behavior	9	−0.06	0.10	−0.59	0.56	[−0.25, 0.14]	0.00%	0.35
IV → Resistance behavior	13	0.05	0.07	0.73	0.47	[−0.08, 0.18]	0.00%	0.54

*Note. k* = number of independent samples cumulated; *b =* unstandardized meta-regression coefficient; *SE* = standard error of the meta-regression coefficient; *z* = Wald z-test statistic for the meta-regression coefficient; *p* = two-tailed probability of statistical significance; 95% CI = confidence interval; *R^2^* = percentage of main effect variance explained by the moderator variable; *Q_M_* = omnibus test statistic for the meta-regression model, assessing whether the moderator explains a significant amount of variance in effect sizes. * *p* < 0.05, ** *p* < 0.01.

## Data Availability

Data are available from the corresponding author upon reasonable request.

## Supplementary Materials

The following supporting information can be downloaded at https://www.mdpi.com/article/10.3390/bs16091474/s1. File S1: PRISMA checklist, which provides the completed PRISMA checklist for the present meta-analysis. File S2: The descriptive overview of studies included, which summarizes the main characteristics of the studies included in the meta-analysis. File S3: Visual assessment of funnel plots and Egger regression asymmetry test, which presents the funnel plots and the results of Egger’s regression asymmetry tests used to assess potential publication bias. References (Amin et al., 2024; Arenas et al., 2023; M. Bai et al., 2025; Califf & Brooks, 2020, 2024; Califf et al., 2020; Chandra et al., 2019; Chang et al., 2024; C. H. Chen & Lee, 2024; Chi et al., 2025; Conroy & O’Leary-Kelly, 2014; Debus et al., 2012; Decataldo & Fiore, 2022; DeLeon, 2021; Ding, 2021; Florkowski, 2019; Galluch et al., 2015; Gercek et al., 2024; Jamieson et al., 2013; Khedhaouria & Cucchi, 2019; Kinnunen et al., 2014; Kruse, 2013; Kurniawan et al., 2022; Lebel et al., 2023; M. Lee & Lim, 2020; Leong et al., 2025; le Roux & Botha, 2021; Lestari et al., 2023; Liu et al., 2024; Mahaptra & Pati, 2018; Mahmoud et al., 2025; Maier et al., 2019; Moore, 2025; Mukhlis et al., 2023; Nelson & Irwin, 2014; Ozer et al., 2021; Pflügner et al., 2021; Reeves, 2024; Salazar-Concha et al., 2022; Sarabadani et al., 2020; Schwabe & Castellacci, 2020; Semaan et al., 2025; Shepherd & Williams, 2018; Simon et al., 2024; Solberg et al., 2020; Song et al., 2024; Tan et al., 2024; Tang et al., 2022; Tarafdar et al., 2010; Techmanska et al., 2024; Teng et al., 2025; H. Wang et al., 2023; Q. Wang & Yao, 2025; Q. Wang & Zhao, 2023; Wu et al., 2024; Yeager et al., 2022; Zainun et al., 2020; C. Zhang et al., 2018; Z. Zhang et al., 2022; Zielonka et al., 2022) are cited in the supplementary materials
