# Supplementary material for: Navigating the Digital Storm: A Systematic Review and Meta-Analysis of Technology-Induced Job Insecurity, Its Outcomes and Moderators
Source: Behav Sci (Basel). 2026 Aug 25;16(9):1474. doi: 10.3390/bs16091474 (PMC13603130; doi:10.3390/bs16091474)
Supplement: Supplementary file 1 [file behavsci-16-01474-s001.zip › behavsci-4298105-supplementary.pdf]

File S1 PRISMA checklist

Section and Topic	Item #	Checklist item	Location where item is reported
**TITLE**			
Title	1	Identify the report as a systematic review.	Page 1
**ABSTRACT**			
Abstract	2	See the PRISMA 2020 for Abstracts checklist.	Page 1
**INTRODUCTION**			
Rationale	3	Describe the rationale for the review in the context of existing knowledge.	Page 1-5
Objectives	4	Provide an explicit statement of the objective(s) or question(s) the review addresses.	Page 5
**METHODS**			
Eligibility criteria	5	Specify the inclusion and exclusion criteria for the review and how studies were grouped for the syntheses.	Page 10-12
Information sources	6	Specify all databases, registers, websites, organisations, reference lists and other sources searched or consulted to identify studies. Specify the date when each source was last searched or consulted.	Page 10
Search strategy	7	Present the full search strategies for all databases, registers and websites, including any filters and limits used.	Page 10
Selection process	8	Specify the methods used to decide whether a study met the inclusion criteria of the review, including how many reviewers screened each record and each report retrieved, whether they worked independently, and if apagelicable, details of automation tools used in the process.	Page 10-12
Data collection process	9	Specify the methods used to collect data from reports, including how many reviewers collected data from each report, whether they worked independently, any processes for obtaining or confirming data from study investigators, and if apagelicable, details of automation tools used in the process.	Page 12
Data items	10a	List and define all outcomes for which data were sought. Specify whether all results that were compatible with each outcome domain in each study were sought (e.g. for all measures, time points, analyses), and if not, the methods used to decide which results to collect.	Page 12
	10b	List and define all other variables for which data were sought (e.g. participant and intervention characteristics, funding sources). Describe any assumptions made about any missing or unclear information.	Page 12
Study risk of bias assessment	11	Specify the methods used to assess risk of bias in the included studies, including details of the tool(s) used, how many reviewers assessed each study and whether they worked independently, and if apagelicable, details of automation tools used in the process.	Page 13
Effect measures	12	Specify for each outcome the effect measure(s) (e.g. risk ratio, mean difference) used in the synthesis or presentation of results.	Datasets
Synthesis methods	13a	Describe the processes used to decide which studies were eligible for each synthesis (e.g. tabulating the study intervention characteristics and comparing against the planned groups for each synthesis (item #5)).	Page 10-12
	13b	Describe any methods required to prepare the data for presentation or synthesis, such as handling of missing summary statistics, or data conversions.	Page 12-13

	13c	Describe any methods used to tabulate or visually display results of individual studies and syntheses.	Page 13
	13d	Describe any methods used to synthesize results and provide a rationale for the choice(s). If meta-analysis was performed, describe the model(s), method(s) to identify the presence and extent of statistical heterogeneity, and software package(s) used.	Page 13
	13e	Describe any methods used to explore possible causes of heterogeneity among study results (e.g. subgroup analysis, meta-regression).	Page 13
	13f	Describe any sensitivity analyses conducted to assess robustness of the synthesized results.	Page 13
Reporting bias assessment	14	Describe any methods used to assess risk of bias due to missing results in a synthesis (arising from reporting biases).	Page 13
Certainty assessment	15	Describe any methods used to assess certainty (or confidence) in the body of evidence for an outcome.	Page 13
**RESULTS**			
Study selection	16a	Describe the results of the search and selection process, from the number of records identified in the search to the number of studies included in the review, ideally using a flow diagram.	Figure. 3
	16b	Cite studies that might apageear to meet the inclusion criteria, but which were excluded, and explain why they were excluded.	N/A
Study characteristics	17	Cite each included study and present its characteristics.	Supplementary material
Risk of bias in studies	18	Present assessments of risk of bias for each included study.	N/A
Results of individual studies	19	For all outcomes, present, for each study: (a) summary statistics for each group (where apageropriate) and (b) an effect estimate and its precision (e.g. confidence/credible interval), ideally using structured tables or plots.	Datasets
Results of syntheses	20a	For each synthesis, briefly summarise the characteristics and risk of bias among contributing studies.	Supplementary material
	20b	Present results of all statistical syntheses conducted. If meta-analysis was done, present for each the summary estimate and its precision (e.g. confidence/credible interval) and measures of statistical heterogeneity. If comparing groups, describe the direction of the effect.	Page 13-16
	20c	Present results of all investigations of possible causes of heterogeneity among study results.	Page 14
	20d	Present results of all sensitivity analyses conducted to assess the robustness of the synthesized results.	Page 14
Reporting biases	21	Present assessments of risk of bias due to missing results (arising from reporting biases) for each synthesis assessed.	Supplementary material
Certainty of evidence	22	Present assessments of certainty (or confidence) in the body of evidence for each outcome assessed.	Page 14
**DISCUSSION**			
Discussion	23a	Provide a general interpretation of the results in the context of other evidence.	Page 16-17
	23b	Discuss any limitations of the evidence included in the review.	Page 18-19
	23c	Discuss any limitations of the review processes used.	Page 18-19
	23d	Discuss implications of the results for practice, policy, and future research.	Page 17-18

OTHER INFORMATION			
Registration and protocol	24a	Provide registration information for the review, including register name and registration number, or state that the review was not registered.	N/A
	24b	Indicate where the review protocol can be accessed, or state that a protocol was not prepared.	N/A
	24c	Describe and explain any amendments to information provided at registration or in the protocol.	N/A
Supageort	25	Describe sources of financial or non-financial supageort for the review, and the role of the funders or sponsors in the review.	Page 20
Competing interests	26	Declare any competing interests of review authors.	Page 20
Availability of data, code and other materials	27	Report which of the following are publicly available and where they can be found: template data collection forms; data extracted from included studies; data used for all analyses; analytic code; any other materials used in the review.	Page 20

File S2. The descriptive overview of studies included

Study	Article Status	Sample Size	DV coded
Alshammary & Hilmi (2024)	P	135	ATJ
Amin et al. (2024)	P	365	BTP
Arenas et al. (2023)	P	931	SE, IS, LAW, PB
Bai et al. (2024)	P	327	ATO, LAW, RB
Bai et al. (2025)	P	310	LAW, RB
Baser et al. (2025)	P	324	BTP, WB
Brougham & Haar (2018)	P	120	ATJ, ATO, LAW, WB
Buzas et al. (2025)	P	361	ATJ, ATO, LAW, PB, RB
Caglar & Taskin (2023)	P	100	WB
Califf & Brooks (2020)	P	416	LAW, WB
Califf & Brooks (2024)	P	392	LAW, WB
Califf et al. (2020)	P	402	SE, ATJ, LAW, WB
Chandra & Srivastava (2019)	P	164	PB
Chang et al. (2024)	P	301	SE, LAW, PB
Chen & Lee (2024)	P	283	PB
Chi et al. (2025)	P	165	RB
Chiu et al. (2023)	P	354	RB
Decataldo & Fiore (2022)	P	415	LAW
DeLeon (2021)	U	150	BTP
Ding (2021)	P	190	ATO, BTP
Erebak & Turgut (2021)	P	243	PB
Fan et al. (2020)	P	191	PB
Ficapal-Cusí et al. (2025)	P	1,187	IS
Florkowski (2019)	P	169	ATJ, IS
Gercek et al. (2024)	P	321	ATJ, PB
Girardi et al. (2024)	P	85	IS
Goethals & Ziegelmayer (2022; Study 1)	P	54	SE, LAW
Goethals & Ziegelmayer (2022; Study 2)	P	112	SE, LAW
Gupta & Banerjee (2025)	P	395	WB
Hampel & Sassenberg (2021)	P	137	RB

Hampel et al. (2022)	P	127	RB
Hur & Shin (2024)	P	301	SE, PB
Issa (2022)	P	63	IS
Jin et al. (2024)	P	349	IS, LAW
Kang et al. (2023)	P	301	IS, PB
Khedhaouria & Cucchi (2019)	P	161	LAW
Kim & Kim (2024)	P	402	SE, LAW, RB
Kim & Lee (2021)	P	620	RB
Kruse (2013)	U	115	BTP
Kurniawan et al. (2022)	U	409	ATJ, ATO, WB
La Torre et al. (2024)	P	1,746	IS, BTP
le Roux & Botha (2021)	P	106	BTP
Lee & Lim (2020)	P	164	SE, ATJ
Leong et al. (2025)	P	500	LAW, BTP, PB
Lestari et al. (2023)	U	160	ATJ, IS
Li et al. (2019)	P	468	WB
Li & Wang (2021)	P	312	BTP
Li & Geng (2025)	P	319	RB
Mahaptra & Pati (2018)	P	133	ATJ, LAW, WB
Mahmoud et al. (2025)	P	403	BTP
Maier et al. (2019; Study 1)	P	126	LAW, BTP
Maier et al. (2019; Study 2)	P	408	LAW, BTP
Mangundu & Mayayise (2023)	P	210	LAW
Marchiori et al. (2020)	P	263	ATJ, ATO
Moore (2025)	U	77	LAW, BTP
Mukhlis et al. (2023)	U	536	ATJ, WB
Olya et al. (2024)	P	319	PB
Ozer et al. (2021)	P	156	PB
Pericleous et al. (2025)	P	270	WB
Pflügner et al. (2021)	P	134	LAW
Pirkkalainen et al. (2019)	P	846	SE, BTP, PB, WB, RB
Reeves (2024)	U	101	LAW
Salazar-Concha et al. (2022)	P	1,047	IS
Sarabadani et al. (2020)	U	188	LAW
Schwabe & Castellacci (2020)	P	10,051	ATJ

Semaan et al. (2025)	P	347	IS
Shadbad & Biros (2022)	P	356	IS, RB
Simon et al. (2024)	P	361	SE, ATJ
Song et al. (2024)	P	378	LAW, BTP, PB
Tan et al. (2024)	P	224	BTP
Tarafdar et al. (2010)	P	233	BTP, PB
Techmanska et al. (2024)	P	187	SE
Teng et al. (2025)	P	554	BTP, PB
Turja et al. (2022)	P	187	ATJ
Urukovicova et al. (2023)	P	692	SE, LAW, BTP
Verma & Singh (2022)	P	486	PB
Walczok & Bipp (2025; Study 1)	P	215	SE
Walczok & Bipp (2025; Study 2)	P	224	SE
Walczok & Bipp (2025; Study 3)	P	233	SE
Wang et al. (2022)	P	264	PB
Wang et al. (2023)	P	235	LAW
Wang et al. (2025)	P	159	PB
Wang & Yao (2025)	P	251	SE, IS, LAW
Wang & Zhao (2023)	P	289	PB
Wu et al. (2024)	P	657	PB
Yang & Jiang (2025)	P	319	PB, WB
Yang et al. (2025)	P	2,814	LAW
Yang et al. (2024)	P	295	BTP, PB
Yin et al. (2024; Study 1)	P	102	SE, PB
Yin et al. (2024; Study 2)	P	191	SE, PB
Zainun et al. (2020)	P	225	PB
Zhang et al. (2022)	P	254	LAW, WB
Zhao et al. (2020)	P	513	BTP, PB, WB, RB
Zhao et al. (2023)	P	359	ATO, RB
Zheng & Zhang (2025)	P	303	IS, LAW
Zielonka et al. (2022)	U	207	ATJ, IS

Note. SE = self-evaluation; *ATJ* = attitudes toward job; *ATO* =attitudes toward organization ; *IS* = immediate strain; *LAW* = long-term affective well-being; *BTP* = basic task performance; *PB* = proactive behavior; *WB* = withdrawal behavior; *RB* = resistance behavior.

File S3. Visual assessment of funnel plots and Egger regression asymmetry test

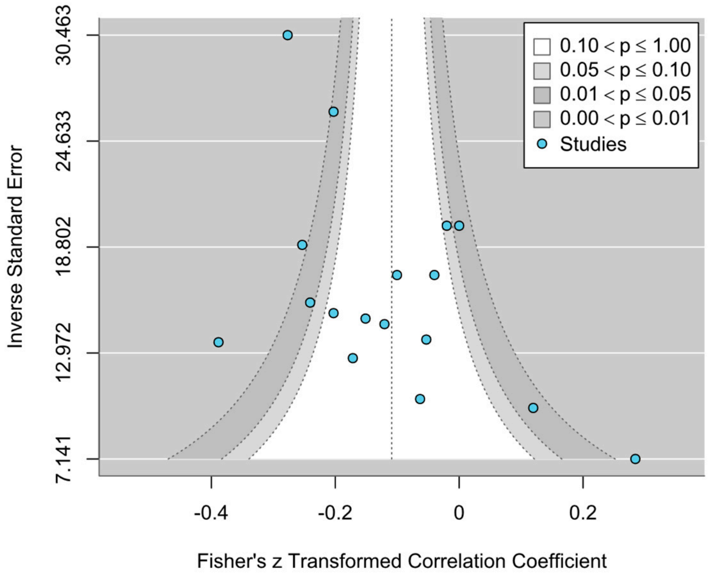

Figure S1. Self-evaluation as dependent variable

Regression test result for funnel plot asymmetry was not significant ($t = 0.37$, $p = 0.714$).

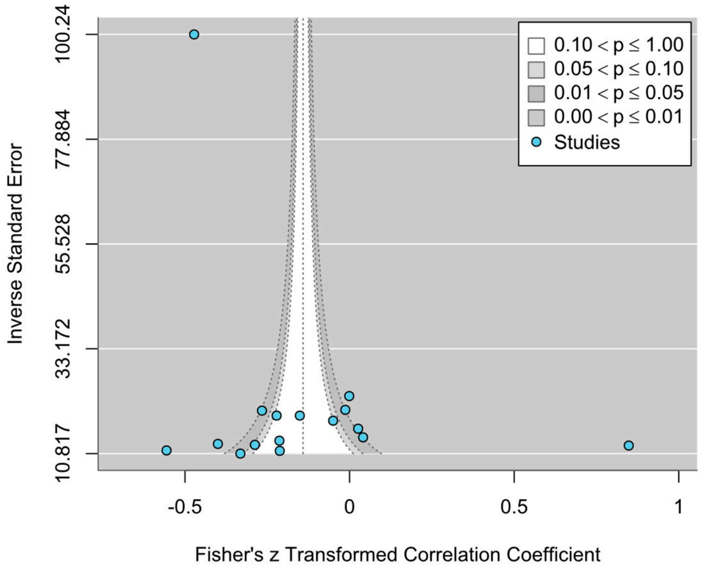

\

Figure S2. Attitudes toward job as dependent variable

Regression test result for funnel plot asymmetry was significant ($t = 3.91$, $p < 0.05$).

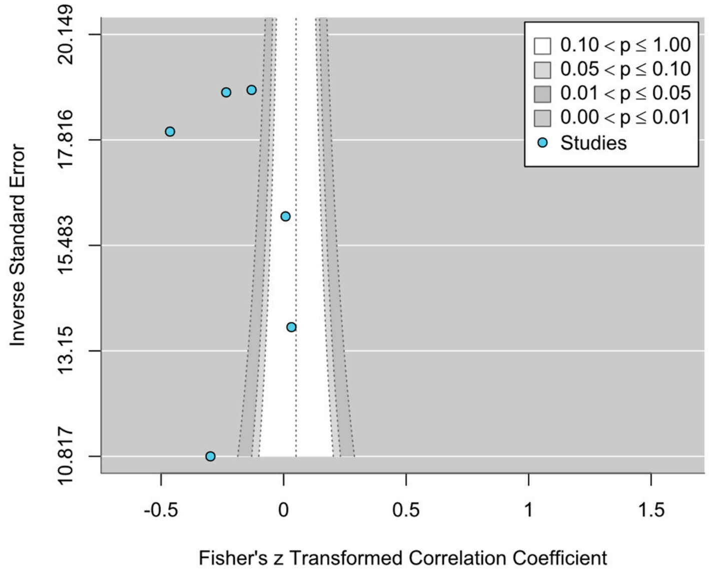

Figure S3. Attitudes toward organization as dependent variable

Regression test result for funnel plot asymmetry was not significant (t = -0.81, p = 0.454).

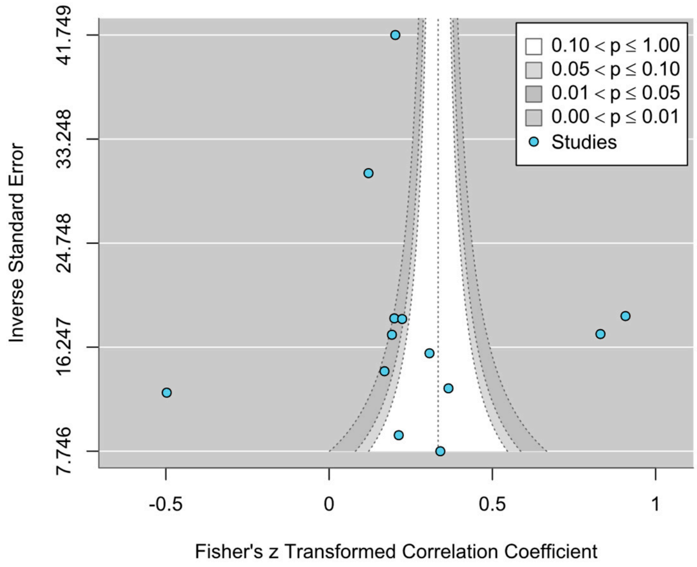

Figure S4. Immediate strain as dependent variable

Regression test result for funnel plot asymmetry was not significant (t = -0.58, p = 0.574).

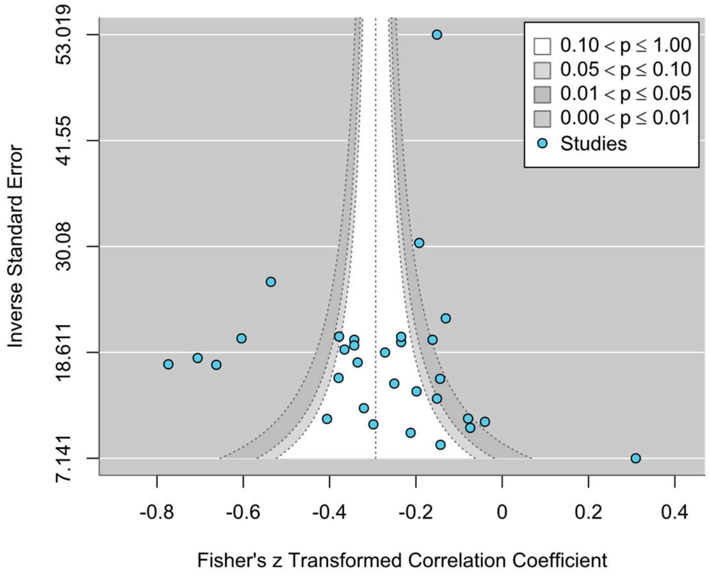

Figure S5. Long-term affective well-being as dependent variable

Regression test result for funnel plot asymmetry was not significant (t = -0.83, p = 0.416).

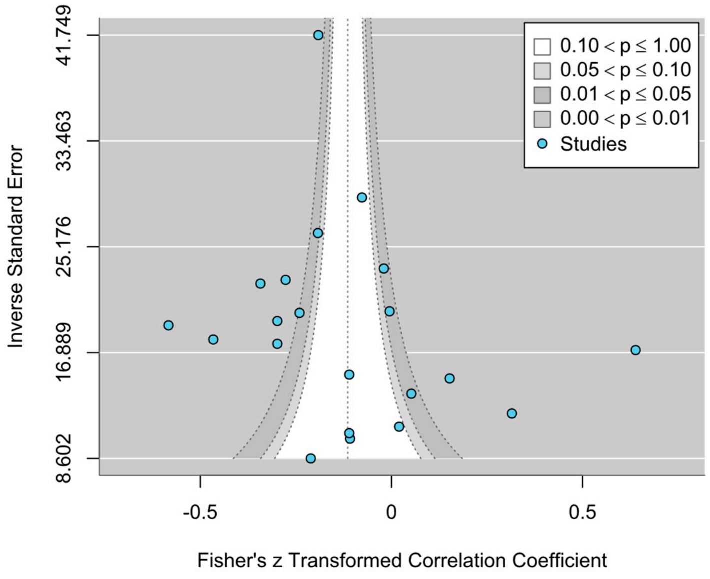

Figure S6. Basic task performance as dependent variable

Regression test result for funnel plot asymmetry was not significant (t = 0.96, p = 0.351).

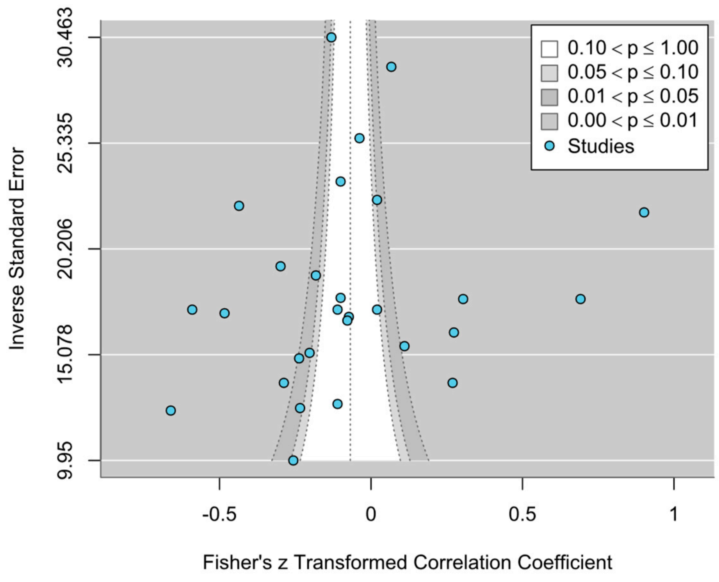

Figure S7. Proactive behavior as dependent variable

Regression test result for funnel plot asymmetry was not significant (t = -0.78, p = 0.442).

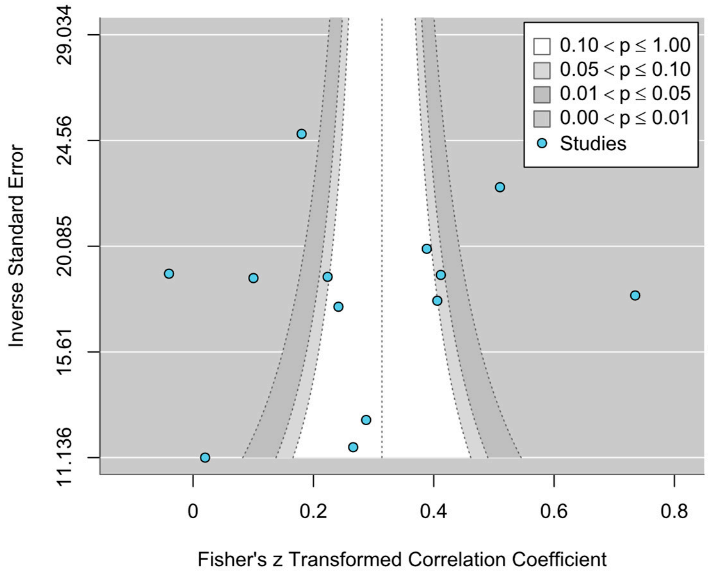

Figure S8. Resistance behavior as dependent variable

Regression test result for funnel plot asymmetry was not significant (t = - 1.45, p = 0.171).

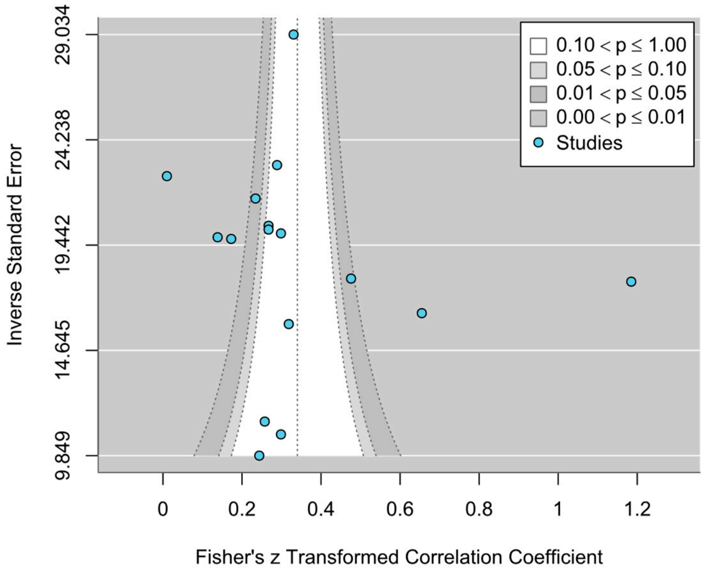

Figure S9. Withdrawal behavior as dependent variable

Regression test result for funnel plot asymmetry was not significant ($t = 0.64$, $p = 0.532$).
